# A Case of Progressively Worsening Dyspnea and Chronic Cough in a Patient With Known Bronchiectasis

**DOI:** 10.1016/j.chest.2026.03.026

**Published:** 2026-08-10

**Authors:** David Eldeiry, Alexander Geyer, Stephen Machnicki

**Affiliations:** a Department of Pulmonary and Critical Care Medicine, Lenox Hill Hospital, Northwell Health, New York, NY; b Department of Radiology, Lenox Hill Hospital, Northwell Health, New York, NY

## Abstract

A 53-year-old Black woman was referred to the pulmonary clinic for progressively worsening exertional dyspnea over the past 3 years and a chronic nonproductive cough. She had a history of childhood asthma, with 2 prior hospitalizations, as well as an unspecified nasal surgery 20 years ago. She was diagnosed with bronchiectasis 10 years ago by her primary care physician and was compliant with airway clearance techniques, including nebulized hypertonic (7%) saline and albuterol-ipratropium and a positive expiratory pressure handheld device. Review of systems was positive for fatigue, seasonal allergies, occasional heartburn, and occasional stiffness of the small joints of the hands. She had a family history of asthma in her cousin and sarcoidosis in her father; her mother had a significant smoking history and was deceased from complications of lung cancer. She had no history of occupational exposures, no pets, and no significant travel history.

## Physical Examination Findings

Examination revealed a middle-aged Black woman in no respiratory distress. Vital signs were as follows: BP, 131/83 mm Hg; heart rate, 82 beats/min; respiratory rate, 12 breaths/min; temperature, 97.9 °F; and peripheral oxygen saturation, 97% on ambient air. General examination did not elicit any abnormal findings; lung sounds were clear bilaterally without crackles or wheezing, she had no evidence of jugular venous distention or lower extremity edema, and skin examination did not reveal sclerodactyly, telangiectasias, or any manifestations of rheumatologic disease.

## Diagnostic Studies

Complete blood count with differential did not identify any abnormal values, and peripheral eosinophils were 0.1 cells/μL. The comprehensive metabolic panel was unremarkable. C-reactive protein was < 3 mg/L. Autoantibodies, including anti-double-stranded DNA, anti-Sjögren-syndrome-related antigen A and B, myeloperoxidase antibodies, myositis-specific and myositis-associated antibodies, anti-centromere, anti-topoisomerase I, rheumatoid factor, and anti-cyclic citrullinated peptide, were all negative. Her antinuclear antibody titer was 1:320 with a speckled pattern.

*Aspergillus* IgG antibodies were negative. Serum immunoglobulin panel was notable for an elevated serum IgE of 585 kU/L. Serum angiotensin-converting enzyme and soluble IL-2 receptor levels were within normal limits. Alpha-1 antitrypsin levels were within the normal range. Sputum cultures showed no growth of bacterial, fungal, or mycobacterial pathogens. Genetic testing was negative for mutations associated with cystic fibrosis and primary ciliary dyskinesia. Sweat chloride testing could not be performed because of insufficient sweat quantity.

Pulmonary function tests revealed mild obstruction with a preserved FEV_1_ (FEV_1_/FVC, 64%; FEV_1_, 1.95 L [90%]), normal lung volumes (total lung capacity, 4.58 L [95%]; FVC, 3.08 L [115%]), and a mildly deceased diffusing capacity (63%).

**Figure 1:**
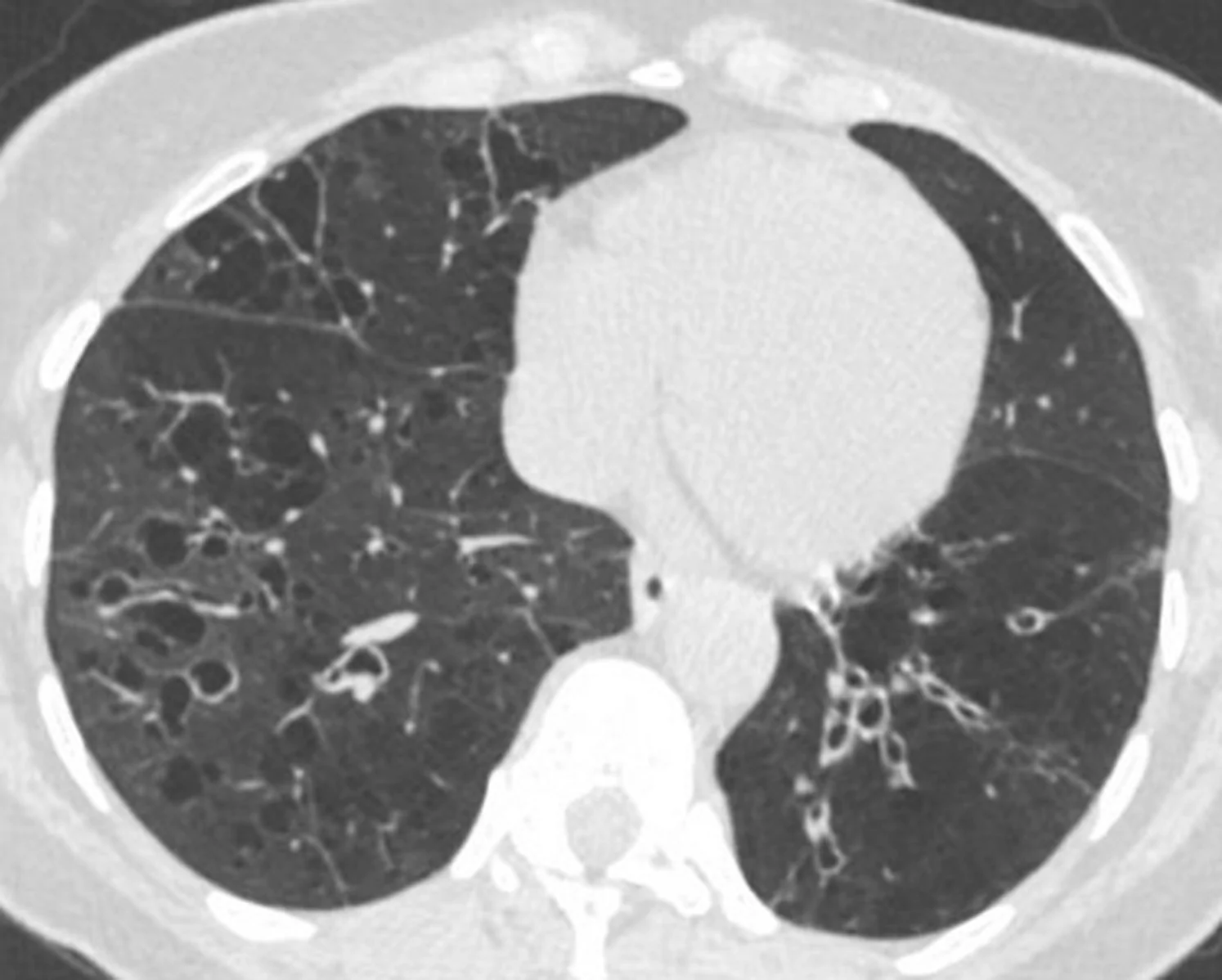
Axial CT scan of cylindrical basilar-predominant bilateral bronchiectasis.

**Figure 2:**
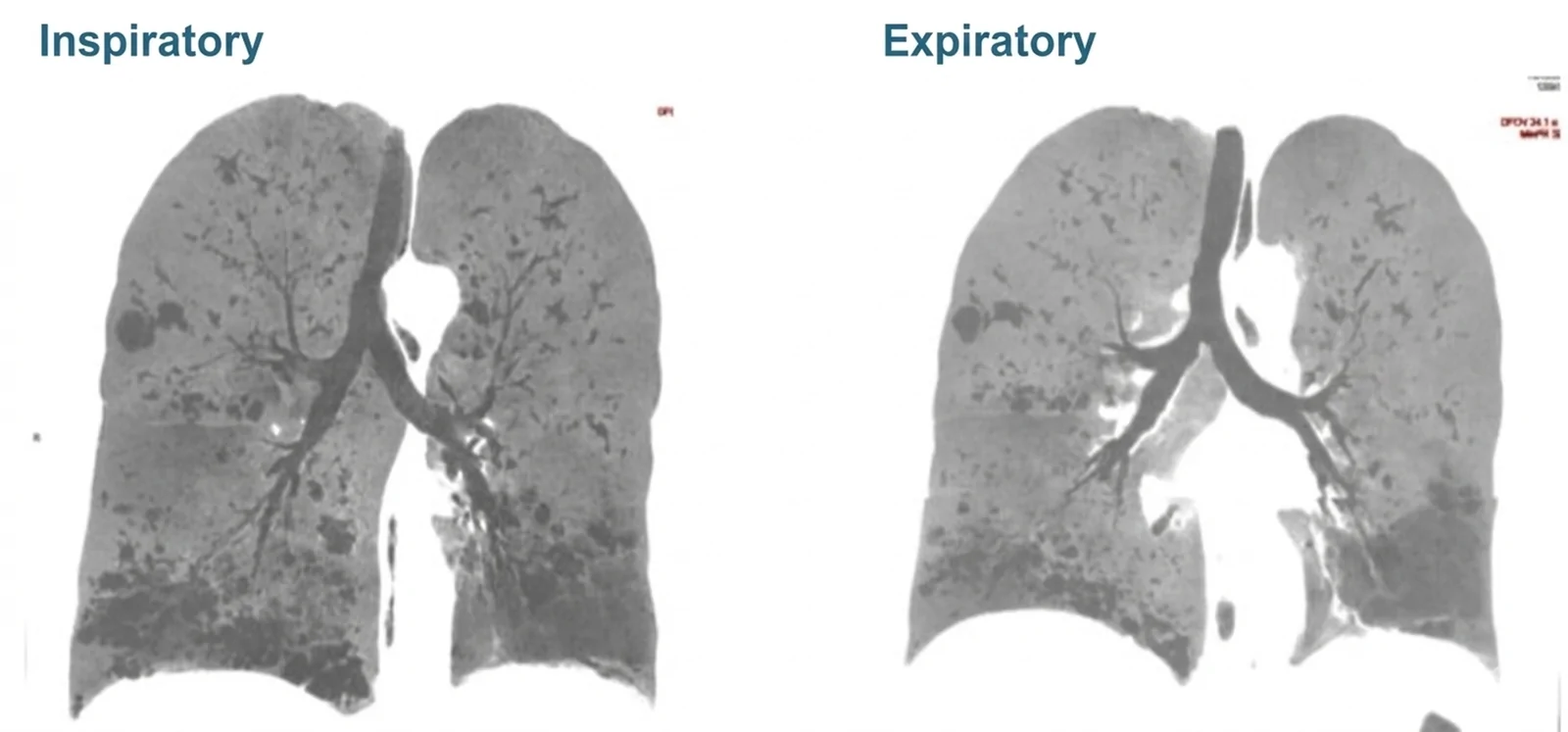
Coronal minimum intensity projections highlighting inspiratory ballooning and expiratory collapse of the bronchiectatic subsegmental bronchi.

The initial CT scan of the chest showed diffuse bilateral cystic bronchiectasis in noncentral airways, primarily in the lower lung fields (Fig 1); these findings were unchanged when compared with a CT scan obtained several years earlier. Dynamic CT chest revealed inspiratory ballooning and expiratory collapse in the regions of bronchiectasis—this was most evident on coronal minimum intensity projections (Fig 2).

*What is the diagnosis?*

*Diagnosis:* William-Campbell syndrome

## Discussion

William-Campbell syndrome (WCS) is a rare cause of congenital cystic bronchiectasis. It was first described in the 1950s in autopsies of pediatric patients with recurrent respiratory infections. It is characterized by defective or absent bronchial wall cartilage in the subsegmental bronchi, leading to distal airway collapse and bronchiectasis formation. It uniquely involves the fourth- to sixth-order bronchial divisions, and the trachea and proximal bronchi are of normal caliber. No specific genes have been identified.

Symptom burden depends on the extent of cartilage maldevelopment or absence. Although most cases present in childhood, the existence of an adult phenotype suggests patients with less severe cartilage deficiency may remain subclinical and undiagnosed for many years. Common symptoms include productive cough, wheezing, and recurrent pneumonia. There is no evidence to suggest that cartilage deficiency occurs outside the lungs.

Typical radiographic features in WCS include a uniform bilateral distribution of cystic bronchiectasis. Dynamic CT scan of the chest has been suggested to best demonstrate the inspiratory ballooning and expiratory collapse of the bronchiectatic segments. Using minimum intensity projections to accentuate low-density structures, such as airways, allows for easier identification of these structural changes and can facilitate diagnosis. Although definitive diagnosis can only be made through lung biopsy revealing deficient cartilaginous plates in affected bronchial segments, it is infrequently done because of a high chance of false-negative results and the increased procedural risk seen in this patient cohort.

Management is primarily supportive. Prophylactic antibiotics for patients with frequent exacerbations, noninvasive positive-pressure ventilation, aggressive airway clearance techniques with nebulized mucolytics and positive expiratory pressure handheld devices, and pulmonary rehabilitation all have been described as part of the management for patients with WCS. Lung transplantation has previously been described in a single patient with WCS; however, data are scarce. Transplantation criteria should likely reflect recommendations for patients with cystic fibrosis, including reduced or rapidly decreasing FEV_1_, increasing exacerbation frequency or exacerbation requiring ICU admission, and refractory/recurrent hemoptysis.

### Clinical Course

The patient is being managed with aggressive airway clearance techniques, with plans to refer to pulmonary rehabilitation. She has not been referred for lung transplantation evaluation because she has had no exacerbations, and her physiologic testing is not grossly abnormal.

## Clinical Pearls

1. *WCS is a rare congenital cause of peripheral cystic bronchiectasis that spares the trachea and central bronchi.*
2. *Adult patients with WCS typically have less severe cartilage deficiency, leading to a longer subclinical undiagnosed disease course.*
3. *Advancements in CT imaging have led to minimum intensity projections and multiplanar volume-rendered reformations becoming exciting new modalities that can characterize airway anatomy and more accurately diagnose rare conditions such as WCS.*
4. *Definitive diagnosis of WCS can only be made through lung biopsy, which is an imperfect and potentially high-risk intervention in this patient population; dynamic CT scan with advanced airway reconstruction provides clinicians an alternative means of obtaining anatomic and functional information through a less-invasive measure.*
5. *Treatment options for WCS are primarily supportive and include antibiotics for frequent exacerbations, aggressive airway clearance techniques, pulmonary rehabilitation, and consideration of lung transplantation in high-risk patients.*

## Financial/Nonfinancial Disclosures

None declared.
